# *In Vivo* Biological Evaluation of Orthodontically Moved Incisors after Replantation

**DOI:** 10.3390/medicina56090421

**Published:** 2020-08-20

**Authors:** Jose de Albuquerque Calasans-Maia, Monica Diuana Calasans-Maia, Maria Bernadete Sasso Stuani, Adriana Terezinha Neves Novellino Alves, Pietro Montemezzi, Carlos Fernando de Almeida Barros Mourão, Julio Pedra e Cal-Neto, Antonio Carlos de Oliviera Ruellas

**Affiliations:** 1 Orthodontics Department, Dentistry School, Universidade Federal Fluminense, Rio de Janeiro 24020-140, Brazil; 2 Oral Surgery Department, Dentistry School, Universidade Federal Fluminense, Rio de Janeiro 24020-140, Brazil; monicacalasans@id.uff.br; 3 Orthodontics Department, Dentistry School, Universidade de São Paulo Ribeirão Preto, Ribeirão Preto 14049-900, Brazil; bernadete@forp.usp.br; 4 Oral Diagnosis Department, Dentistry School, Universidade Federal Fluminense, Rio de Janeiro 24020-140, Brazil; aterezinha@gmail.com; 5 Private Practice, 24128 Bergamo, Italy; m.montemezzi@libero.it; 6 Post-Graduation Program, Dentistry School, Universidade Federal Fluminense, Rio de Janeiro 24020-140, Brazil; mouraocf@gmail.com; 7 Orthodontics Department, Instituto de Saúde de Nova Friburgo, Universidade Federal Fluminense, Rio de Janeiro 28625-650, Brazil; juliocalneto@yahoo.com.br; 8 Orthodontics Department, Dentistry School, Universidade Federal do Rio de Janeiro, Rio de Janeiro 21941-617, Brazil; aruellas@umich.edu

**Keywords:** avulsed tooth, replanted tooth, orthodontic movement, in vivo, rats

## Abstract

*Background and Objectives:* There is still considerable controversy regarding the possibility of submitting replanted teeth to orthodontic movement (OM). The purpose of the present study was to evaluate the tissue response after orthodontic movement on replanted teeth. *Materials and Methods:* Sixty Wistar rats were randomly assigned to four groups (*n* = 15): G1, replantation without OM after 30 days; G2, replantation with OM after 30 days; G3, replantation without OM after 60 days, and G4, replantation with OM after 60 days. The maxillary left central incisors were extracted and the teeth were stored in milk media. After 30 min, the teeth were replanted and fixed with non-rigid immobilization. All specimens were observed after 30 and 60 days of replantation and then subdivided into two subgroups (with OM or without OM). The animals were euthanized after seven days of the OM started, and the maxillary bone blocks were processed for histological evaluation. *Results:* The histological results showed periodontal ligament repair in both periods studied without OM; however, ankylosis and root resorption was seen in all orthodontically moved teeth. *Conclusions:* The orthodontic movement did not favor tissue response in all replanted teeth, regardless of the experimental periods.

## 1. Introduction

The early loss of an incisor can cause severe psychological and functional damage such as aesthetic loss, occlusion, and poor speech since avulsions involving permanent teeth are commonly seen in youth, where root development is incomplete [1]. Immediate dental replantation is considered the most appropriate clinical procedure for achieving a successful tooth avulsion treatment, although it is not always possible [2].

The sequelae regarding the replantation of the avulsed teeth involve surface resorption, inflammatory resorption, replacement resorption, and dentoalveolar ankylosis [3,4,5,6,7]. Avulsion of one or more teeth requires emergency care by the dentist [8], where the first choice is the possibility of replanting the avulsed tooth to restore esthetics and function [9,10].

Some previous studies have suggested that there is an association of dental trauma and malocclusion [11,12,13,14,15,16,17]. Increased overjet (more than 4 mm), poor labial sealing, short upper lip, Angle Class II malocclusion, and mouth breathing can represent significant characteristics predisposing upper incisor traumatism, and it was the most common factors related to dental trauma [16]. Patients with these characteristics are also candidates for orthodontic treatment and often look for treatment with a history of prior dental trauma.

As accidental dental trauma commonly occurs in children, some may need orthodontic treatment for malocclusion correction soon. In the literature, clinical studies report that after four or five months of follow-up, slightly or moderately traumatized periodontal ligament can be orthodontically moved with a similar prognosis as the non-traumatized tooth [18]. However, there are no pre-clinical or clinical studies in the literature with teeth that were severely traumatized (tooth avulsion) and then submitted to orthodontic treatment. A previous study concluded that orthodontic treatment purposes must be reviewed when the patient shows evident root resorptions [18]. These authors advised a thorough examination of the root outline, observing the occurrence of any root resorptions, surface concavities, and malformations, since teeth with these characteristics may be severely reabsorbed during treatment. 

A significant number of animal studies seeking successful experimental treatments for tooth avulsion is available in the literature [19,20,21,22,23,24], but not with orthodontic movement. For the first time, the present study aimed to evaluate the tissue response of orthodontic movement on avulsed teeth after 30 min of replantation. 

## 2. Materials and Methods

Animal experiments and breeding were performed under conditions approved by the Institutional Review Board of the Universidade de São Paulo–Ribeirão Preto (Protocol number 05.1.1174.53.0), in compliance with the National Institute of Health (NIH) Guide for Care and Use of Laboratory Animals and with Brazilian legislation on animal use. This study was conducted according to the guidelines of the 3R’s Program (Reduction, Refinement, Replacement) and reported according to the ARRIVE [25] guidelines (Animal Research: Reporting of In Vivo Experiments) with regard to relevant items and Planning Research and Experimental Procedures on Animals: Recommendations for Excellence (PREPARE) [26].

The sample size calculation was performed using the G Power software by the ANOVA test (Analysis of Variance).

After the calculus, a sample size of at least 14 rats in each group was required to achieve 80% power at a significance level of 5%. This value was adjusted for 5% attrition and was allocated around 15 rats per group [27] with a total of 60 animals (Table 1). Sixty male Wistar rats (*Rattus norvegicus*, Albinus) weighing 250–300 g was used. The animals’ age was approximately 90 days, and they were obtained from the bioterium of the Universidade de São Paulo Dentistry School at Ribeirão Preto. 

During the experiment, the animals were kept in individual cages (*n* = 2) specially prepared for this purpose. The rats were daily fed with solid ground rations (Guabi Nutrilabor, Mogiana Alimentos, São Paulo, Brazil) and water was provided ad libitum during the experiment. Vitamin C was supplemented by adding 20 mg of ascorbic acid to the water supply, and the room temperature was kept between 16 °C and 20 °C. A standard 12-h light–dark cycle was adopted to establish an optimal metabolic cycle. The supervision of animal care, diet, and pre- and post-operative fasting were conducted by a veterinary with experience in rodents.

Before the procedures, the animals received general anesthesia with ketamine hydrochloride 25 mg/kg (Virbac, Jurubatuba, SP, Brazil) and xylazine hydrochloride 1 mg/Kg (FortDodge, São Cristovão, RJ, Brazil), both intraperitoneal [28].

The left maxillary incisors of all animals were extracted in a non-traumatic technique. The dental papilla and the enamel organ were removed from each extracted tooth by using a #11 scalpel blade (Bard Parker, Caledonia, MI, USA), according to previous studies that aimed to interrupt the continuous growing of the tooth [29,30]. Then, they were immediately stored in milk for 30 min. 

After this period of time, each tooth had its most coronal portion wrapped with gauze, and the pulp tissue was extirpated through a retrograde via with a #15 Hedström file (25 mm, Maillefer, Dentsply, MI, USA). The root canal was irrigated with 0.9% sodium chloride solution, and a syringe with a 25 × 27 mm needle was used for aspirating the residues and dried with absorbent paper points. The same root canal was filled with saturated calcium hydroxide paste. Immediately after the root filling, the teeth were replanted into their respective socket [31].

**Figure 1:**
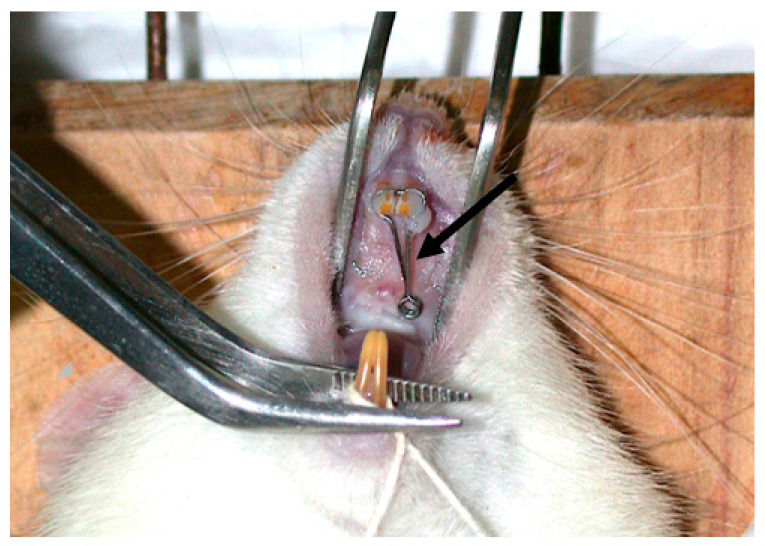
Orthodontic appliance performed with 0.016” cross-sectioned round stainless wire in order to promote inclined dental movement and a diastema between both upper incisors.

According to the purpose of the present study, the animals were randomly divided into four groups consisting of 15 animals each, as follows:

After replantation, all animals received a single intramuscular 20,000 UI Penicillin G Procaine dose (Wycillin R 400,000 UI–Fontoura-Wyeth, São Paulo, SP, Brazil). The orthodontic movement was achieved by using an appliance made of 0.016” cross-sectioned round stainless wire in order to promote inclined dental movement and a diastema between both upper incisors (Figure 1). A double coil spring of 1.5 mm diameter was medially positioned 7 mm from the upper incisors in an anteroposterior sense and 1 mm from the bony palate. Two incisor-oriented parallel wires were separated from each other in 0.5 mm and surrounded the distal faces toward the vestibular direction.

A cavity was made between the two upper incisors and parallel and transversal horizontal grooves along the teeth axis. Grooves were also made buccally, medially, and distally in the central incisors at the interdental papilla apex and the palatine surface. Both hole and grooves were made by using a 0.5 spherical diamond drill mounted on a counter angle and adapted to a low-speed electric motor. The grooves enabled both better adjustment and attachment of the orthodontic appliance. The coil spring was attached to the incisors with a bonding composite (TPH Spectrum, Dentsply). A dynamometer was used for assessing and confirming the activated spring at 0.5 N. The inserted coil springs were checked daily in terms of attachment, stability, and corrections to prevent injury to the animal’s buccal mucous membrane. 

After 37 and 67 days, the animals were euthanized with an overdose of general anesthesia, and the bone block containing the replanted tooth was removed, fixed in 10% neutral formaldehyde solution for one week, and then submitted to the histological process, cut into 5 µm, and stained with Hematoxylin and Eosin (HE) for histological and histomorphometric evaluation.

### 2.1. Histomorphometric Analysis

**Figure 2:**
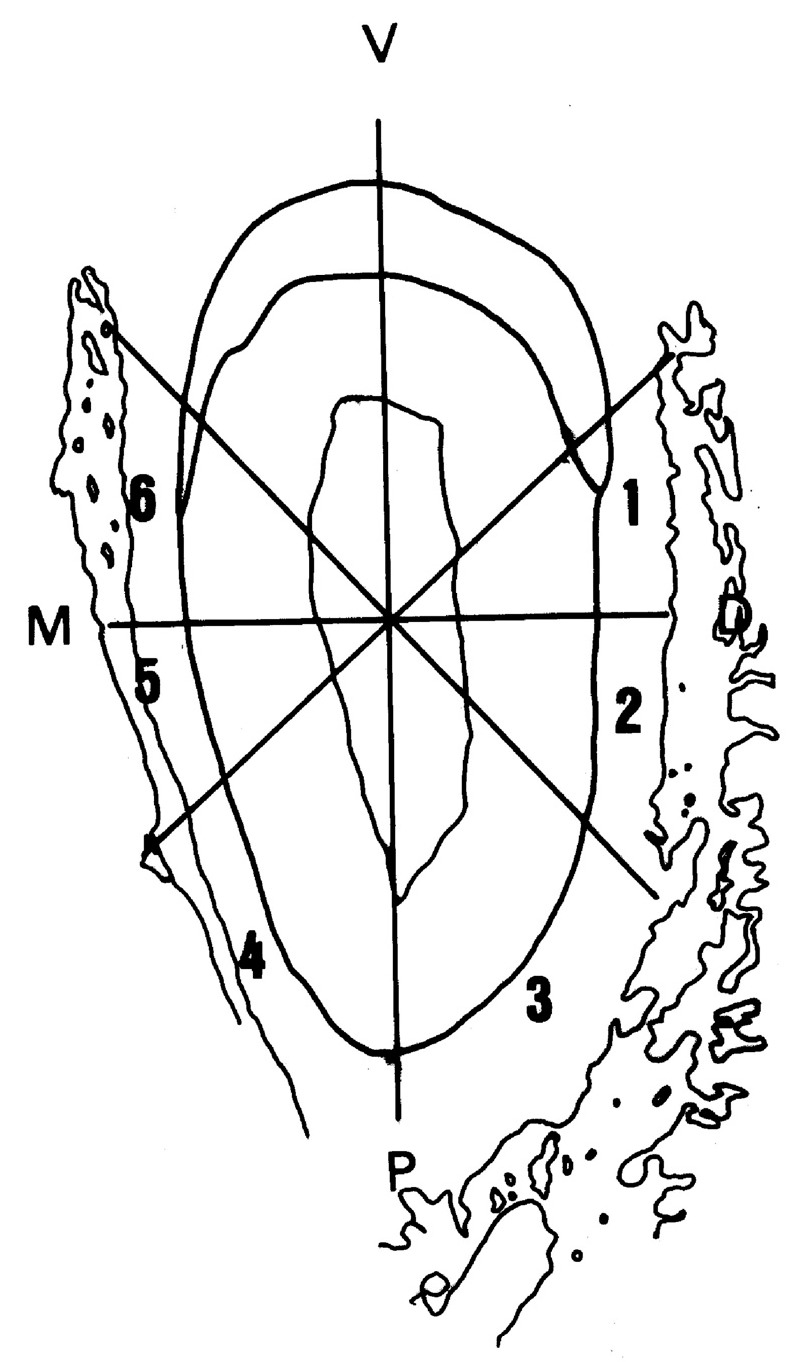
The six root areas evaluated the cross-sectioned incisor root. A grid mask was superimposed to the geometric center of pulp cavity to evaluate the biological response of the periodontium according to the experimental period.

The histomorphometric evaluation of the root surface was performed according to the modified method described by Andreasen [32]. A grid consisting of four lines intersecting each other was positioned over the cross-sectioned incisor root photomicrograph. The center of the grid superimposed the geometric center of the pulp cavity, and the vertical line superimposed the greatest buccal-lingual diameter of the root. In this manner, histological changes in the supporting periodontium could be evaluated through the intersections of the four lines with the root surface. A blinded observer performed the histological and histomorphometric evaluation (Figure 2). 

During the orthodontic movement, the moved teeth presented inclination according to pressure (regions 1, 2, and 3) and tension (regions 4, 5, and 6), totalizing 45 areas in the pressure side and 45 areas in the tension side of each of the groups.

The periodontal response characteristics were classified according to the following periodontal parameters:

(a) Repair: small resorption areas repaired by cement neoformation;
(b) Root resorption: inflammatory resorption areas with the presence of an inflammatory infiltrate in addition to the multinucleated cells; and
(c) Ankylosis: deposition of bone tissue juxtaposed to the cementum layer.

Cross-sectioned incisors and the middle portion of the supporting periodontium were used for statistical analysis. Cervical and apical regions were not used as the cervical is frequently traumatized during the extraction, and the apical had the dental papilla removed.

### 2.2. Statistical Analysis

Descriptive statistics were performed to evaluate the data obtained in the histomorphometric analysis. The association between histological evaluation and the experimental groups was statistically analyzed with Fisher’s exact test, with a significance level of 5%.

## 3. Results

The results showed that the orthodontic movement did not favor the periodontal ligament repair in both experimental periods. The histological evaluation of both experimental periods showed better results for the tension side (areas 4, 5, and 6) compared to the pressure side (areas 1, 2, and 3). Three regions of each animal/side were evaluated (*n* = 45). These histological findings are shown in Table 2 (pressure side) and Table 3 (tension side). 

By applying Fisher’s exact test to the histological evaluations, it was observed that the results were statistically significant in all groups without orthodontic movement regardless of the post-replantation periods of 30 or 60 days. In terms of percentage, better results regarding the periodontal ligament repair were observed in those groups following 60 days of replantation (Table 4).

**Figure 3:**
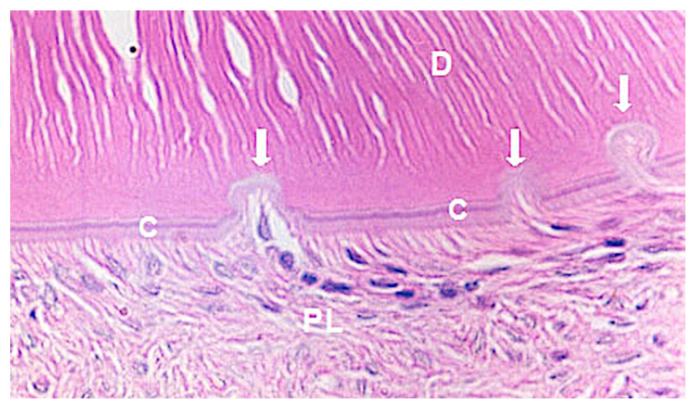
Photomicrographic of the repaired area of the periodontal ligament. Arrows indicate inactive resorption lacunae with neoformation of cement; C-cement; D-dentin; PL-periodontal ligament. Magnification: 40X; Stain: HE. HE: Hematoxilin and Eosin.

**Figure 4:**
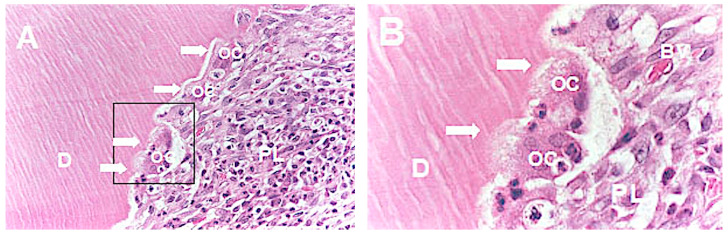
Photomicrographs of tooth presenting root resorption. (**A**) Inflammatory infiltrate in the periodontal ligament and inflammatory resorption with osteoblasts in the Howship’s lacunae. Arrows indicate active resorption lacunae (HE–40X). (**B**) Active osteoblasts in detail. Arrows indicate ruffled border (HE–100X). OC-osteoclasts; D-dentine; PL-periodontal ligament; BV-blood vessel.

**Figure 5:**
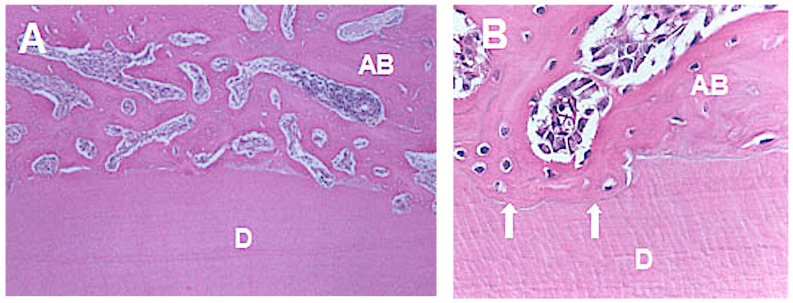
Photomicrograph of tooth presenting ankylosis. (**A**) ample view of ankylosis (HE–40X). (**B**) replacement resorption in detail with bone tissue formation (HE–100X). AB-alveolar bone; D-dentine.

The group without orthodontic movement and after 60 days of replantation (Figure 3) presented 71.1% of periodontal ligament repair (Figure 3). Comparing the pressure and tension sides concerning the experimental periods, 80% of root resorption on the pressure side was observed after 30 days of replantation (Figure 4A,B) and 62.2% after 60 days, and 20% of ankylosis (Figure 5A,B) after 30 days versus 37.8% after 60 days.

## 4. Discussion

An adequate moment for submitting traumatized teeth to orthodontic movement is not known. Dental trauma promotes damage to the cementoblast layer as well as the extended areas without cementoblasts. The surrounding osteoblasts can also replace the cementoblasts and assume their phenotype and functions efficiently [4].

The orthodontic movement may represent another injury for those previously traumatized because the controlled areas consisting of cementoblast-like osteoblasts can be replaced by osteoremodeling units (clasts, osteoblasts, and macrophages) and start an ankylosis process [32]. The extra-alveolar period and the storage medium in which the tooth was kept before replantation is an important factor in allowing the survival and regeneration of the damaged periodontium [33]. Replacement of avulsed teeth is time-dependent, the earlier the tooth is replaced, the better the prognosis.

In this study, all replanted teeth were stored for 30 min in whole milk. A previous study concluded that whole milk and soymilk favored the periodontal repair process of replanted teeth in rats when associated with laser photobiomodulation [2]. Another study speculated that 20 min of extraoral dry time is detrimental to the periodontal ligament cells as 60 or 90 min of extraoral dry time [33].

However, a recent study showed that the immediate application of mild and controlled orthodontic forces was not detrimental to the periodontal healing of teeth replanted after 20 min of extraoral dry time, although no significant improvement in periodontal healing was observed [34].

The histological evaluation of all the replanted teeth without orthodontic movement had better results regarding periodontal ligament repair than the replanted teeth with orthodontic movement; this result corroborates the findings by Brin et al. [35]. This study observed a higher incidence of root resorption on the pressure side; these results were also observed in previous studies [4,9,22].

Concerning the tension side, the root regions had 64.4% of root resorption after 30 days versus 53.3% after 60 days, and 35.6% of ankylosis after 30 days versus 46.7% after 60 days, thus suggesting a higher incidence of root resorption for both experimental periods, despite the lower 60-day values. Ankylosis was also observed in a more significant number of root regions after 60 days. Similar results were found in the literature [5]. Therefore, according to the data described above, the more extended post-replantation period is indicative of better periodontal conditions for orthodontic movement in cases of severe traumatism, although longevity was not indicated. 

The lack of periodontal ligament repair in all studied groups after either 30 or 60 days of replantation does not recommend the start of orthodontic treatment. However, it was seen in the literature that the immediate application of mild and controlled orthodontic forces was not detrimental to the periodontal healing of the replanted teeth [34]. Parameters determining the most suitable moment for submitting traumatized teeth to orthodontic movement can be found in the literature: for slight or moderate dental traumatism, 3–6 months of observation should be considered, and if periodontal conditions are reasonable, the orthodontic force can be applied; for severe dental traumatism, one year of observation should be considered; if periodontal conditions are clinically and radiographically normal, the orthodontic force can be used [36,37,38]. Finally, orthodontic treatment is not recommended until the periodontal recovery of the replanted tooth is complete. 

This study used a pre-clinical model with rodents. It is known that the incisors of rats are continuously growing teeth and that for this reason, the results of the study could not be extrapolated to humans. However, previous studies have shown that removing the tooth papillae and enamel organ of the rat’s tooth would interrupt its growth, making it more similar to the human tooth’s behavior. It should be considered that the results obtained in groups without OM demonstrated a tendency of tissue repair, and therefore OM should be delayed. 

The clinician should know that traumatized teeth with signs of root resorption before the orthodontic treatment are at a high risk of increased root resorption as a result of orthodontic movement [39,40]. 

Clinical studies should be conducted with the purpose of elaborating guidelines on the proper timing of orthodontic force application after short- and long-term replantation. These guidelines would be helpful in guiding orthodontists who are initiating orthodontic treatment in patients with a history of replanted teeth.

## 5. Conclusions

Based on the results found in this study, it can be suggested that:

The orthodontic movement did not favor the periodontal ligament repair in both experimental periods. 

After 30 days of replantation, the orthodontic movement did not interfere with the root resorption; however, after 60 days, the orthodontic movement favored the root resorption. 

The orthodontic movement favored the ankylosis after 30 and 60 days of replantation. 

In the group without orthodontic movement, the periodontal ligament repair increased along the experimental periods. The root resorption decreased from 30 to 60 days, and the ankylosis was observed only after 60 days of replantation. 

## Figures and Tables

**Table 1:** Sixty rats divided into four experimental groups according to the experimental periods after replantation and subsequently orthodontic movement.

Groups	*n*	Orthodontic Movement	Euthanasia
1	15	----	30 days after onset of experiment
2	15	30 days after reimplantation	7 days after orthodontic movement
3	15	----	60 days after onset of experiment
4	15	60 days after reimplantation	7 days after onset of orthodontic movement

**Table 2:** Histological evaluation of the pressure side of all groups.

Groups	Total		Histological Evaluation of the Pressure Side					
			Repair		Root Resorption		Ankylosis	
	*n*	%	*n*	%	*n*	%	*n*	%
1 (30 days-without movement)	45	100	15	33.3	30	66.7	0	0.0
2 (30 days-with movement)	45	100	0	0.0	36	80.0	9	20.0
3 (60 days-without movement)	45	100	33	73.3	0	0.0	12	26.7
4 (60 days-with movement)	45	100	0	0.0	28	62.2	17	37.8
Total	180	100	48	26.7	94	52.2	38	21.1

**Table 3:** Histological evaluation of the tension side of all groups.

Groups	Total		Histological Evaluation in the Tension Side					
			Repair		Root Resorption		Ankylosis	
	*n*	%	*n*	%	*n*	%	*n*	%
1 (30 days-without movement)	45	100	14	31.1	31	68.9	0	0.0
2 (30 days-with movement)	45	100	0	0.0	29	64.4	16	35.6
3 (60 days-without movement)	45	100	31	68.9	0	0.0	14	31.1
4 (60 days-with movement)	45	100	0	0.0	24	53.3	21	46.7
Total	180	100	45	25	84	46.7	51	28.3

* represents a statistically significant difference between groups at same experimental time point.

**Table 4:** Effect of the orthodontic movement on the histological evaluation after 30 min of replantation (regions 1–6)**.**

Post-Replantation Period	Histological Evaluation	Orthodontic Movement				Total		*p*-Value
		No		Yes				
		*n*	%	*n*	%	*n*	%	
30 days	Repair	29	32.2	0	0.0	29	16.1	<0.001
	No repair*	61	67.8	90	100	151	83.9	
	Total	90	100	90	100	180	100	
60 days	Repair	64	71.1	0	0.0	64	35.6	<0.001
	No repair*	24	26.7	90	100	116	64.4	
	Total	90	100	90	100	180	100	
